# Communication inequalities in the COVID-19 pandemic: socioeconomic differences and preventive behaviors in the United States and South Korea

**DOI:** 10.1186/s12889-023-16211-8

**Published:** 2023-07-05

**Authors:** Woohyun Yoo, Yangsun Hong, Sang-Hwa Oh

**Affiliations:** 1 Department of Media and Communication & Institute of Social Sciences, Incheon National University, Incheon, South Korea; 2 Department of Communication and Journalism, University of New Mexico, Albuquerque, NM USA; 3 Charles H. Sandage Department of Advertising, College of Media, University of Illinois at Urbana-Champaign, Champaign, IL USA

**Keywords:** Communication inequalities, COVID-19, Socioeconomic characteristics, Comparative analysis, Structural influence of communication

## Abstract

**Background:**

Communication inequalities are important mechanisms linking socioeconomic backgrounds to health outcomes. Guided by the structural influence model of communication, this study examined the intermediate role of health communication in the relationship between education, income, and preventive behavioral intentions during the COVID-19 pandemic in the United States and South Korea.

**Methods:**

The data were collected through two online surveys conducted by two professional research firms in the US (April 1–3, 2020) and South Korea (April 9–16, 2020). To test the mediating role of health communication, as well as the hypothesized relationships in the proposed model, we performed a path analysis using Mplus 6.1.

**Results:**

In analyzing survey data from 1050 American and 1175 Korean adults, we found that one’s socioeconomic positions were associated with their intentions to engage in COVID-19 preventive behaviors through affecting their health communication experiences and then efficacious beliefs. Differences in education and income were associated with willingness to engage in preventive behaviors by constraining health communication among people with low levels of education and income. The findings showed notable differences and some similarities between the US and South Korea. For example, while income was positively associated with health communication in both US and South Korea, education was only significantly related to health communication in US but not in South Korea.

**Conclusions:**

This study suggests health communication strategies such as choice of communication channels and messages to promote intention for COVID-19 prevention behaviors in particular consideration of individual differences in socioeconomic positions in countries with different cultural features. Pubic policies and health campaigns can utilize the suggestions to promote efficacy and preventive behavioral intention during early pandemics.

## Background

The coronavirus disease 2019 (COVID-19) pandemic led to socially disadvantaged populations being more vulnerable to risks and threats related to this public health crisis, especially during the early stages of the pandemic [1–3]. The disparities are exacerbated for people facing socioeconomic disadvantages due to inadequate access to health care, low health literacy, high-exposure occupations, and household overcrowding [4].

When a public health emergency occurs, disadvantaged societal groups may experience particular constraints, such as limited opportunities to access health information resources, challenges in understanding such information, and difficulties in adopting recommended preventive behaviors. Scholars have articulated that such communication inequalities function as mechanisms linking individuals’ positions in social structural hierarchies to health outcomes [5–7].

Especially during a pandemic, individual differences in socioeconomic status (SES), such as education and income, can cause severe communication inequalities, which, in turn, influence individuals’ health beliefs and ultimately, their preventive practices [6–8]. Guided by the structural influence model (SIM) of communication [5], this study examines the intermediate role of communication as a pathway in which individuals’ positions in socioeconomic hierarchies lead to them taking preventative measures against COVID-19. In addition, the present research elaborates on the role of communication inequalities by comparing the processes and impacts of health communication between the United States (US) and South Korea.

These two countries were selected for the following reasons. First, there were notable differences in government responses to the outbreak and the patterns of COVID-19 morbidity and mortality during the early stages of the pandemic [9, 10]. While most states and cities had state-at-home orders and there was slow rollout of COVID-19 testing in the US [11], the South Korean government took an “agile-adaptive approach” that encouraged social distancing and voluntary quarantine and offered massive, rapid preventative COVID-19 testing [9, 12]. Second, the two countries had varying cultural, social, and environmental contexts, which are related the governmental preventive measures and also affect people’s communication and their health decision making. For example, as a highly collectivist society, South Korea has a tight culture which expects citizens to follow social norms and social rules in their behaviors (e.g., mask wearing). In this context, individuals prefer communicating with personal contacts or close ties when it comes to a sensitive, controversial, or polarized issue like COVID-19 to avoid a potential conflict or punishment for being deviant from the social norms. In contrast, US is known as an individualist society that has a relatively loose culture with weak social norms and rules that individuals are expected to follow [35, 36].

### Socioeconomic inequalities and preventive behaviors against infectious diseases

Not everyone faces the same burdens related to global health risks like the COVID-19 pandemic. Socioeconomic inequalities profoundly influence the incidence and treatment of such communicable diseases, ultimately exacerbating inequalities in infection and mortality by SES. Specifically, those with the lowest positions in socioeconomic hierarchies and with limited power have an increased likelihood to encounter a variety of difficulties and challenges prior, during, and after the COVID-19 pandemic [13–15].

People with a lower socioeconomic status might be quarantined in overcrowded settlements and workplaces, making physical distancing impossible and self-isolation challenging, thus resulting in an increased risk of spreading or contracting COVID-19 [16]. During the early period of the COVID-19 pandemic, about 25% of US workers transitioned to remote work to prevent the spread of COVID-19 infection. Essential workers had a greater risk of exposure to COVID-19 than workers employed by non-essential businesses [17].

Unequal access to information about preventive strategies and resources helping individuals adopt preventive behaviors is another factor at play in this phenomenon [14, 18]. In times of public health emergencies, economic hardship can make lower-SES individuals’ access to health facilities and healthcare services more difficult [13]. Thus, pre-existing socioeconomic inequalities may deteriorate health outcomes. Previous studies found that people with lower education and income levels tended to have higher rates of non-compliance with COVID-19 safety precautions [19–21].

### The structural influence model and the mediating role of communication

**Fig. 1:**
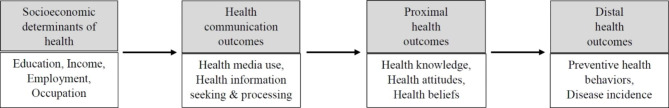
The structural influence model (SIM) of communication

Health communication inequalities refer to differences in the capacity to access, process, and act upon health information via several types of media, such as news media, social media, and instant messaging services [22]. According to the SIM of communication [5, 23], inequalities in health communication at least partially mediate the relationship between socioeconomic conditions and health outcomes. As shown in Fig. 1, health communication presents a critical pathway through which socioeconomic characteristics influence proximal health factors, such as health knowledge, attitudes, beliefs, and distal outcomes, ultimately yielding health inequalities [24].

The media are an effective and popular conduit for health communication. For the novel COVID-19 pandemic, people wanted to learn new social rules and build their perceptions of new norms regarding the preventive behaviors through the media, interpersonal interactions, and observation of others [25, 26]. In the early stages of the pandemic, however, people had limited opportunities to interact with and observe others since social distancing and facial masks were strongly enforced in public spaces. When the use of one particular communication channel is constrained or when the information obtained from a communication source is not sufficient, people tend to use other channels as substitutes to access the information they need [27, 28]. Thus, given the loss of interpersonal connections due to social distancing, mediated communication channels, such as the mass media, social media, and instant messaging services, became major sources for individuals to learn social rules and build perceptions and beliefs related to COVID-19 prevention.

Furthermore, drawing on theoretical frameworks related to communication and persuasion, such as protective motivation theory [29], the health belief model [30], and the extended parallel process model [31], scholars have elucidated the psychological mechanisms through which exposure to media messages predicted preventive behaviors during the COVID-19 pandemic. Given the prevalence of uncertainty and ambiguity in the context of novel diseases and the recommended preventive behaviors, previous studies have highlighted the significant role of efficacious beliefs. For instance, Chung and Jones-Jang [32] found that those who obtained COVID-19 information from traditional media showed heighted efficacy perceptions, which, in turn, promoted their intentions to follow the recommendations for COVID-19 preventive measures. Truong et al. [33] also showed that both mass media use and social media use were positively associated with perceived self-efficacy, which, in turn, increased coping appraisal, finally resulting in greater engagement in COVID-19 preventive behaviors.

Building on these findings, it is expected that exposure to media messages about COVID-19 can influence individuals’ efficacious beliefs, which would, in turn, affect their willingness to adopt the recommended preventive behaviors. Therefore, taken together with the SIM model and considering the discussion above, we hypothesize that the associations between one’s socioeconomic status and intention to engage in COVID-19 preventive behaviors will be serially channeled through media use for health communication and efficacious beliefs. Among the various socioeconomic factors, this study focuses on education and income.

#### H1

The association between education and preventive behavioral intention will be mediated through health communication (news media use, social media use, and use of instant messaging services) and efficacy (self-efficacy and response efficacy).

#### H2

The association between income and preventive behavioral intention will be mediated through health communication (news media use, social media use, and use of instant messaging services) and efficacy (self-efficacy and response efficacy).

### Cultural differences in the mechanisms to predict engagement in COVID-19 preventive practices

Based on cultural tightness-looseness theory in the cultural dimension, Gelfand et al. [34] demonstrated that countries with a tighter culture have lower numbers of COVID-19 cases and lower mortality rates than countries with looser cultures. Tightness-looseness theory [35] posits that each country varies in their degrees of cultural tightness-looseness, which is represented by two constructs: the strength of social norms and the tolerance of deviant behavior. Countries with tighter cultures have relatively “strong social norms and a low tolerance of deviant behavior,” while countries with looser cultures tend to have “weak social norms and a high tolerance of deviant behavior” [34]. This cultural difference influences psychological and behavioral outcomes through multilevel processes from distal factors, like ecological and historical aspects (e.g., the environment and population density) and socio-political institutions (e.g., the government, policies, and the media), to micro-level factors (e.g., everyday experiences and situational constraints). These factors interact with each other, shaping the strength of societal norms and the level of tolerance of deviance in a society [34, 36].

Specifically, people learn about new social rules and the potential punishment for not following them from news media, social media posts, or interpersonal interactions, which, in turn, affect their psychological responses to the new rules and their behavioral decision-making regarding the recommended preventions. However, the procedures and outcomes differ by country due to cultural differences.

In this sense, this study explores whether the mediating role of health communication in the relationship between socioeconomic factors and compliance with early-pandemic precautionary measures to prevent COVID-19 infection was different between countries with varying levels of cultural tightness-looseness. To examine this question, we compare the US (a country with looser culture) and South Korea (a country with tighter culture) based on Gelfand and colleagues’ distinction [36]. Given that there is not much empirical evidence for cultural differences during the COVID-19 pandemic, we pose the following two research questions:

#### RQ1

Do the mediating effects of health communication in the relationship between education and preventive behavioral intention differ between the US and South Korea?

#### RQ2

Do the mediating effects of health communication in the relationship between income and preventive behavioral intention differ between the US and South Korea?

## Methods

### Procedure and participants

The data were collected through two online surveys conducted by two professional research firms in the US (April 1–3, 2020) and South Korea (April 9–16, 2020). Participants were recruited from Qualtrics in the US and Macromill Embrain in South Korea. We set quotas for age, gender, household income (US), and region (South Korea) based on census data. When the panelists visited the online survey site, a more detailed description of the survey and a consent form were provided, along with statements guaranteeing voluntary participation, anonymity, and confidentiality. Following approval from the University of Illinois Urbana-Champaign Institutional Review Board (IRB No. 20,696), two versions of the survey questionnaire (in English and Korean) were developed. Participants who provided incomplete or insincere responses to any of the questions were excluded, resulting in a final sample of 1050 participants (55.5% response rate) from the US and 1175 participants (59.8% response rate) from South Korea. Missing data were negligible since full data were collected from participants with no missingness.

### Measures

#### Exogenous variables

We operationalized socioeconomic status with two indicators. Education was categorized into five levels based on the highest level of education degree achieved (1 = less than high school to 5 = graduate degree). Income was measured as the total household income earned over a 12-month period and categorized into nine groups (US: 1 = less than $10,000 to 9 = $150,000 or more, South Korea: 1 = less than 12 million Korean won to 9 = 96 million Korean won or more).

Age, gender, political orientation, and COVID-19 risk perception were considered as exogenous variables that were linked to all the endogenous variables. Age was measured as a continuous variable by asking participants to report their age. Gender was assessed as a binary variable (0 = male, 1 = female). Political orientation was measured using a single item on a 7-point Likert-type scale (1 = strongly conservative to 7 = strongly liberal), with higher scores indicating more liberal views. COVID-19 risk perception was assessed using a 7-point Likert-type scale (1 = strongly disagree to 7 = strongly agree) and participants were asked to rate their agreement with six statements (e.g., “I believe that COVID-19 can be a serve health problem to me”) adapted from previous research [37].

#### Endogenous variables

News media use was measured using a 7-point Likert-type scale (1 = never to 7 = very often), asking respondents how often they saw, heard, or read information about COVID-19 from (1) television news, (2) newspapers, (3) radio news, (4) online news sites, and (5) news portal sites (US: Cronbach’s *α* = 0.74, South Korea: Cronbach’s *α* = 0.63).

Social media use was assessed using a 7-point Likert-type scale (1 = never to 7 = very often), asking participants how often they saw, heard, or read information about COVID-19 from (1) Facebook, (2) YouTube, (3) Twitter, and (4) Instagram (US: Cronbach’s *α* = 0.78, South Korea: Cronbach’s *α* = 0.76).

Use of instant messaging services was measured using a 7-point Likert-type scale (1 = never to 7 = very often), asking participants how often they saw, heard, or read information about COVID-19 through (1) WhatsApp (US) or Kakao Talk (South Korea), (2) Facebook Messenger, and (3) other types of instant messaging services (US: Cronbach’s *α* = 0.87, South Korea: Cronbach’s *α* = 0.70).

Self-efficacy was measured using three items (e.g., “I will be able to avoid getting infected with COVID-19”) [38] on a 7-point Likert-type scale (1 = strongly disagree to 7 = strongly agree) (US: Cronbach’s *α* = 0.71, South Korea: Cronbach’s *α* = 0.77).

Response efficacy was assessed using two items (e.g., “Engaging in preventive measures recommended by the government and the health organizations prevents infection with COVID-19”) [38] on a 7-point Likert-type scale (1 = strongly disagree to 7 = strongly agree) (US: Cronbach’s *α* = 0.86, South Korea: Cronbach’s *α* = 0.90).

COVID-19 preventive behavioral intention was measured with four items (e.g., “I will wash my hands often with soap and water for at least 20 seconds”) [39] on a 7-point Likert-type scale (1 = strongly disagree to 7 = strongly agree) (US: Cronbach’s *α* = 0.81, South Korea: Cronbach’s *α* = 0.87).

#### Analytic procedure

**Fig. 2:**
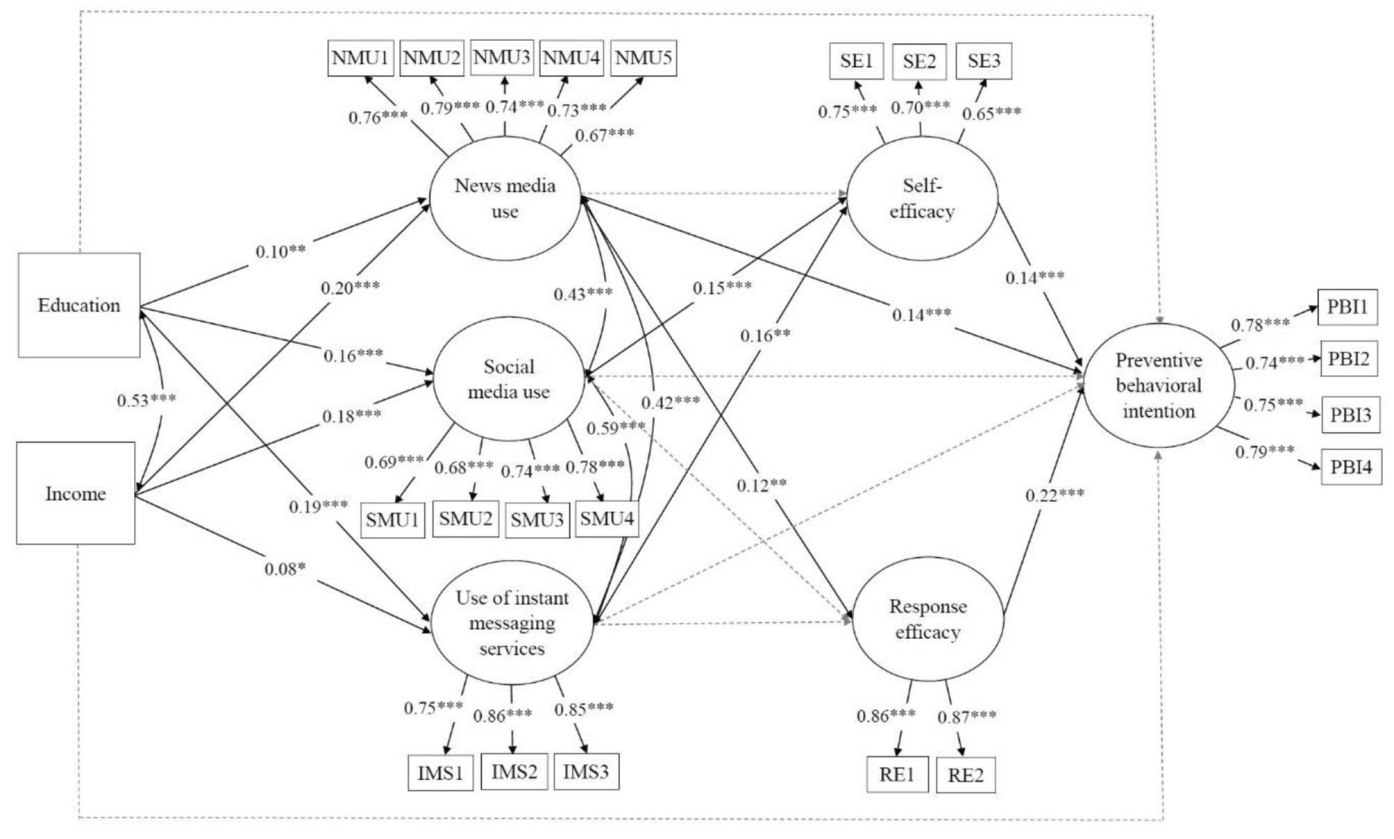
Structural equation model for the US sample (*N* = 1075). All the coefficients are standardized. Solid black lines represent statistically significant relationships, while dotted gray lines indicate nonsignificant relationships. *p < 0.05, **p < 0.01, ***p < 0.001

**Fig. 3:**
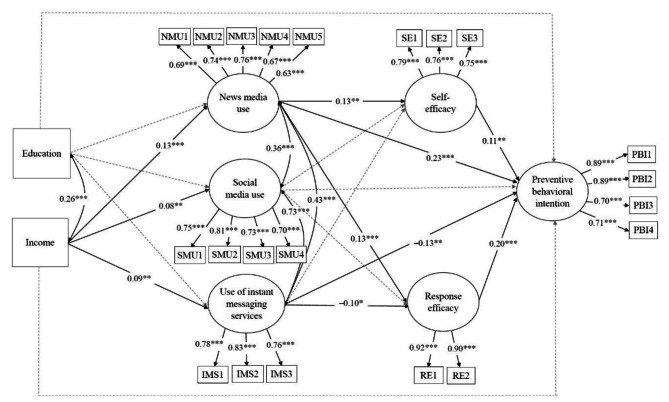
Structural equation model for the South Korea sample (*N* = 1150). All the coefficients are standardized. Solid black lines represent statistically significant relationships, while dotted gray lines indicate nonsignificant relationships. *p < 0.05, **p < 0.01, ***p < 0.001

Before fitting the structural equation model to examine direct and indirect association in the proposed model, we performed multigroup confirmatory factor analysis (MGCFA) to verify the factor structure of our measurement model. The measurement model consisted of the six latent factors with 21 observed indicators (5 items for news media use, 4 items for social media use, 3 items for use of instant messaging services, 3 items for self-efficacy, 2 items for response efficacy, and 4 items for preventive behavioral intention). MGCFA was assessed with chi-square statistics, root mean square error of approximation (RMSEA), comparative fit index (CFI), Tucker-Lewis index (TLI), and standardized root mean square residual (SRMR). Based on the model fit criteria [40], the MGCFA model was well fitted across the two groups, χ^2^ = 4051.74, df = 378, *p* < 0.001, RMSEA = 0.03, CFI = 0.97, TLI = 0.94, and SRMR = 0.01 (for factor loadings, see Figs. 2 and 3).

After fitting the measurement model, we conducted a multigroup structural equation modeling (MGSEM) to test the hypothesized relationships in the proposed model and compare the association differences between the United States and South Korea. We used the Mplus 6.1 program with a maximum likelihood estimator with robust standard errors (MLR) to handle non-normally distributed data. We first constrained all parameters in the MGSEM to be equal in both countries, but the overall model fit was statistically significantly worse (∆ χ^2^ = 400.67, ∆*df* = 49, *p* < 0.001), which lead to the rejection of the null hypothesis that the associations are the same across the two groups. According to the model fit criteria [40], the fully unconstrained MGSEM was a good fit for both groups, χ^2^ = 5069.52, df = 576, *p* < 0.001, RMSEA = 0.04, CFI = 0.98, TLI = 0.92, and SRMR = 0.03. Therefore, the unconstrained MGSEM model performed well and enabled the testing of the statistical significance of the causal relationships among the variables. The indirect effects of socioeconomic characteristics on preventive behavioral intentions were examined using bootstrapping procedures with 10,000 bootstrap samples, generating bias-corrected 95% confidence intervals for the indirect impacts. All analyses controlled for age, gender, political orientation, and risk perception.

## Results

### Descriptive statistics and bivariate correlations

The participants in the US sample had a mean age of 46.22 years, with 51.0% being male (*N* = 536), while those in the South Korean sample had an average age of 44.27 years, with 49.9% being male (*N* = 586). South Korean participants reported higher levels of liberal orientations compared to American participants (US: *M* = 4.15, *SD* = 1.85, vs. South Korea: *M* = 4.34, *SD* = 1.22, *t* = -2.829, *p* < 0.01). Conversely, American participants had a higher perception of risks associated with COVID-19 compared to South Korean participants (US: *M* = 5.73, *SD* = 1.31, vs. South Korea: *M* = 5.28, *SD* = 1.12, *t* = 8.633, *p* < 0.001). With regard to the highest level of education, the majority of US respondents (49.0%) had completed high school education, followed by a bachelor’s degree (19.0%) and a graduate degree (11.0%). Meanwhile, the majority of South Korean respondents (55.2%) held a bachelor’s degree, followed by a high school diploma (18.2%) and an associate’s degree (15.4%). The median household income in the US sample ranged from $40,000 to under $50,000, while in the South Korean sample it ranged from 48 million to under 60 million Korean won ($35,810-$44,762). Tables 1 and 2 display the means, standard deviations, and bivariate correlations among the model variables.

**Table 1:** Bivariate correlations among key variables (United States)

	1	2	3	4	5	6	7	8	9	10	11	12
1	1.00											
2	-0.02	1.00										
3	-0.18***	0.04	1.00									
4	0.14***	-0.11**	0.15***	1.00								
5	0.05	0.39***	0.12***	0.04	1.00							
6	-0.03	0.28***	0.05	0.03	0.53***	1.00						
7	-0.12***	0.10**	0.13***	0.29***	0.23***	0.28***	1.00					
8	-0.45***	0.19***	0.19***	0.15***	0.28***	0.31***	0.53***	1.00				
9	-0.32***	0.16***	0.15***	0.19***	0.26***	0.22***	0.52***	0.70***	1.00			
10	-0.18***	0.14***	0.04	-0.04	0.19***	0.18***	0.23***	0.33***	0.32***	1.00		
11	-0.02	-0.04	0.09**	0.44***	0.05	0.09**	0.28***	0.21***	0.19***	0.32***	1.00	
12	0.09**	-0.11***	0.05	0.44***	0.08*	0.7*	0.28***	0.14***	0.13***	0.19***	0.43***	1.00
*M*	46.22	1.49	4.15	5.73	2.66	4.85	4.57	3.54	3.19	4.86	5.81	6.16
*SD*	17.19	0.50	1.85	1.31	1.23	2.53	1.53	1.84	2.05	1.29	1.24	1.06

(1) Age; (2) Gender; (3) Political orientation, (4) Risk perception; (5) Education; (6) Income; (7) News media use; (8) Social media use; (9) Use of instant messaging services; (10) Self-efficacy; 11. Response efficacy; 12. Preventive behavioral intention

* *p* < 0.05, ** *p* < 0.01, *** *p* < 0.001

**Table 2:** Bivariate correlations among key variables (South Korea)

	1	2	3	4	5	6	7	8	9	10	11	12
1	1.00											
2	-0.01	1.00										
3	-0.13***	0.06	1.00									
4	0.06	0.02	-0.02	1.00								
5	0.05	-0.15***	0.01	-0.02	1.00							
6	0.02	0.002	0.04	0.01	0.26***	1.00						
7	0.11***	-0.01	0.05	0.24***	0.09**	0.15***	1.00					
8	-0.07*	-0.04	0.09**	0.09**	0.02	0.09**	0.37***	1.00				
9	0.09**	-0.04	0.03	0.13***	0.05	0.09**	0.46***	0.72***	1.00			
10	-0.01	-0.03	0.14***	0.01	0.03	0.06*	0.17***	0.14***	0.13***	1.00		
11	0.10***	0.16***	0.27***	0.22***	-0.03	0.07*	0.15***	-0.01	-0.01	0.42***	1.00	
12	0.06*	0.21***	0.09**	0.33***	0.07*	0.08**	0.27***	-0.02	-0.02	0.21***	0.37***	1.00
*M*	44.27	1.50	4.34	5.28	3.56	5.20	4.83	3.24	3.41	4.59	5.46	6.03
*SD*	13.04	0.50	1.22	1.12	0.93	2.15	1.00	1.44	1.39	1.03	1.03	0.80

(1) Age; (2) Gender; (3) Political orientation, (4) Risk perception; (5) Education; (6) Income; (7) News media use; (8) Social media use; (9) Use of instant messaging services; (10) Self-efficacy; 11. Response efficacy; 12. Preventive behavioral intention

* *p* < 0.05, ** *p* < 0.01, *** *p* < 0.001

### Multigroup structural equation modeling analysis

Table 3 presents the results of a multigroup structural equation modeling (MGSEM) analysis after controlling for covariates. Education and income did not have direct relationships with preventive behavioral intention in either country. Instead, education and income were significantly associated with health communication. As shown in Fig. 2, in the US sample, education was positively related to news media use (*γ* = 0.10, *p* < 0.01), social media use (*γ* = 0.16, *p* < 0.001), and use of instant messaging services (*γ* = 0.19, *p* < 0.001). Furthermore, income was positively associated with news media use (*γ* = 0.20, *p* < 0.001), social media use (*γ* = 0.18, *p* < 0.001), and use of instant messaging services (*γ* = 0.08, *p* < 0.05). In the South Korea sample, income also had a positive relationship with news media use (*γ =* 0.13, *p* < 0.001), social media use (*γ =* 0.08, *p* < 0.01), and use of instant messaging services (*γ =* 0.09, *p* < 0.01). However, there were no significant associations between education and health communication constructs (see Fig. 3).

Regarding the impact of health communication, in the US sample, news media use was positively associated with response efficacy (*β =* 0.12, *p* < 0.01) and preventive behavioral intention (*β =* 0.14, *p* < 0.001). Social media use and use of instant messaging services were also positively related to self-efficacy (*β =* 0.15, *p* < 0.001, *β =* 0.16, *p* < 0.01, respectively). Self-efficacy and response efficacy had positive links with preventive behavioral intention (*β =* 0.14, *p* < 0.001, *β =* 0.22, *p* < 0.001, respectively). In the South Korea sample, only news media use was positively associated with self-efficacy (*β =* 0.13, *p* < 0.01), response efficacy (*β =* 0.13, *p* < 0.001), and preventive behavioral intention (*β =* 0.23, *p* < 0.001). In contrast, the use of instant messaging services was negatively associated with response efficacy (*β =* -0.10, *p* < 0.05) and preventive behavioral intention (*β =* -0.13, *p* < 0.01). Additionally, self-efficacy and response efficacy had positive relationships with preventive behavioral intention (*β =* 0.11, *p* < 0.01, *β =* 0.20, *p* < 0.001, respectively).

**Table 3:** Direct relationships among exogenous and endogenous variables

	United States							South Korea					
	News media use	Social media use	Use of instant messaging services	Self-efficacy	Response efficacy	Preventive behavioral intention		News media use	Social media use	Use of instant messaging services	Self-efficacy	Response efficacy	Preventive behavioral intention
Age	-0.16***	-0.48***	-0.35***	-0.04	-0.02	0.08**		0.10***	-0.07*	0.08**	-0.01	0.12***	0.01
Gender (Male = 0)	0.03	0.08**	0.08**	0.03	-0.03	-0.09**		-0.01	-0.05	-0.05	-0.04	0.13***	0.18***
Political orientation	0.04	0.05	0.03	-0.03	0.001	-0.01		0.06	0.09**	0.04	0.13***	0.28***	0.01
Risk perception	0.30***	0.21***	0.23***	-0.10**	0.39***	0.30***		0.24***	0.09**	0.12***	-0.03	0.20***	0.24***
Education	0.10**	0.16***	0.19***	0.08*	-0.02	-0.04		0.05	-0.004	0.02	-0.01	-0.03	0.01
Income	0.20***	0.18***	0.08*	0.03	0.04	0.03		0.13***	0.08**	0.09**	0.04	0.05	0.03
News media use				0.07	0.12**	0.14***					0.13**	0.13***	0.23***
Social media use				0.15**	0.08	0.04					0.05	-0.02	-0.04
Use of instant messaging services				0.16**	-0.003	-0.07					0.04	-0.10*	-0.13**
Self-efficacy						0.14***							0.11**
Response efficacy						0.22***							0.20***
*R* ^2^	0.19***	0.37***	0.24***	0.15***	0.22***	0.30***		0.09***	0.03**	0.03**	0.05***	0.17***	0.28***

* *p* < 0.05, ** *p* < 0.01, *** *p* < 0.001

H1 proposed that there would be an indirect association between education and preventive behavioral intention, with health communication and efficacy acting as mediators. H2 suggested a similar indirect relationship between income and preventive behavioral intention, also mediated by health communication and efficacy. H1 and H2 were partially supported. Table 4 shows that significant indirect effects of education and income on preventive behavioral intention in both countries, confirming the mediation of health communication and efficacy.

For the US sample, the results of the mediation analysis showed that the indirect links from education to preventive behavioral intention were significantly mediated by news media use (standardized indirect effect = 0.014, *p* < 0.05), social media use and self-efficacy (standardized indirect effect = 0.003, *p* < 0.05), and use of instant messaging services and self-efficacy (standardized indirect effect = 0.004, *p* < 0.05). Similarly, the indirect associations between income and preventive behavioral intention were mediated by news media use (standardized indirect effect = 0.028, *p* < 0.001), news media use and response efficacy (standardized indirect effect = 0.005, *p* < 0.05), and social media use and self-efficacy (standardized indirect effect = 0.004, *p* < 0.05).

The mediation analysis for the South Korean sample revealed that income was indirectly linked to COVID-19 preventive behavioral intention via news media use (standardized indirect effect = 0.03, *p* < 0.001), news media use and self-efficacy (standardized indirect effect = 0.002, *p* < 0.05), news media use and response efficacy (standardized indirect effect = 0.003, *p* < 0.05), and use of instant messaging services (standardized indirect effect = -0.011, *p* < 0.05). However, there was no significant indirect association between education and preventive behavioral intention through health communication and efficacy.

RQ1 and RQ2 aimed to investigate whether there were differences in the mediating effects of health communication on the relationships between socioeconomic factors (education and income, respectively) and preventive behavioral intention between the US and South Korea. The results showed that health communication mediated the relationship between education and preventive behavioral intention in the US, but not in South Korea, which addressed RQ1. However, with respect to RQ2, health communication mediated the relationships between income and preventive behavioral intentions in both the US and South Korea. Additionally, the mediating roles of instant messaging services were significantly different in the US and South Korea. Specifically, the use of instant messaging services mediated the positive associations between income and preventive behavioral intentions in the US sample, while it mediated the negative relationships between income and preventive behavioral intentions in the South Korean sample.

**Table 4:** Significant indirect relationships between education, income and COVID-19 preventive behavioral intention

United States							*Estimate*	*SE*
Education	→	News media use	→	Preventive behavioral intention			0.014*	0.006
Education	→	Social media use	→	Self-efficacy	→	Preventive behavioral intention	0.003*	0.001
Education	→	Use of instant messaging services	→	Self-efficacy	→	Preventive behavioral intention	0.004*	0.002
Income	→	News media use	→	Preventive behavioral intention			0.028***	0.008
Income	→	News media use	→	Response efficacy	→	Preventive behavioral intention	0.005*	0.002
Income	→	Social media use	→	Self-efficacy	→	Preventive behavioral intention	0.004*	0.002
South Korea							*Estimate*	*SE*
Income	→	News media use	→	Preventive behavioral intention			0.03***	0.008
Income	→	News media use	→	Self-efficacy	→	Preventive behavioral intention	0.002*	0.001
Income	→	News media use	→	Response efficacy	→	Preventive behavioral intention	0.003*	0.001
Income	→	Use of instant messaging services	→	Preventive behavioral intention			-0.011*	0.005

*p < 0.05, ***p < 0.001.

## Discussion

This study elucidates that one’s socioeconomic position is associated with one’s experience of health communication regarding COVID-19 through mediated communication channels. We also found that the associations between socioeconomic position and intention to adopt precautionary measures were serially mediated by the experience of health communication and efficacious beliefs. The findings further show notable differences with regard to the varying cultural, social, and environmental contexts related to people’s responses to the pandemic between the US and South Korea, while some similarities were also identified.

In the US sample, household income was an important predictor of using news media, social media, and instant messaging services to obtain COVID-19 information in the US. Likewise, one’s level of education had a positive relationship with health communication. While Americans used news media for COVID-19 (*M* = 4.57) more than social media (*M* = 3.54) and instant messaging services (*M* = 3.19), the association of education with news media use was relatively weaker than those with social media use and instant messaging services. The results imply that people with higher education may prefer their social circles as sources of information during the health crisis, and thus they used social media and instant messaging more than news media for COVID-19 information. Education institution offers a chance for people to build social capital including construction of diverse social network and access to and ability to use valuable resources. Meanwhile, in case of household income, the relationship with news media use was stronger than those with social media use and instant messaging services. That is, people with higher household income used institutionalized sources like news media for COVID-19 information more than their social contracts such as social media and instant messaging service.

On the other hand, income was associated with using news media, social media, and instant messaging services for COVID-19 information in South Korea, and the association was stronger with news media use, similar to the results observed in US. However, education was not significantly related to health communication in South Korea. In other words, one’s level of degree attained was not associated with use of news media, social media, and instant messaging services for COVID-19 information in the context of South Korea. It may be related to negative skewness of the education variable. About 81% of respondents had more than associate’s degree in South Korea, which is about double of US sample (38%). This can be also explained by cultural difference. In countries with tighter culture, people tend to be vigilant about a new phenomenon and pay attention to information and resources that they can learn social norms and rules regardless of education. It is because when a society faces collective threats, strict social rules (e.g., mask wearing in public spaces) are easily institutionalized, and punishment for deviances (e.g., expression of hatred toward non-maskers) are enforced and allowed as ways to overcome the threats collectively [35, 36]. Access to material resources, such as communication devices and an Internet connection, is essential for utilizing media channels to obtain COVID-19 information. Therefore, in South Korea, people’s economic conditions might predict their level of engagement in health communication through media channels.

These results indicate that the differences in socioeconomic positions have less influence on engaging in health communication via mediated channels in a country with a tighter culture than in a country with a looser culture. Population density is one of the macro-level factors that distinguish cultures [41]. More densely populated societies tend to have tighter social norms and people are more likely to be sensitive to social norms and more willing to quickly adapt new social rules to avoid potential punishments [42–44]. Compared to people in the US, which is ranked as the 161st country with a high population density, people living in South Korea, the country ranked 21st for high population density among 213 countries [45], may be more actively seeking normative information. It is highly likely that South Koreans quickly adjusted to the new social rules surrounding COVID-19 prevention and were quite willing to avoid getting punished for engaging in any deviant behaviors in public. Thus, the findings imply that the social expectations and normative pressures in a tighter culture reduced the influence of socioeconomic inequalities on health communication in South Korea.

Our findings also shed light on the mediating role of health communication. The associations between socioeconomic positions and intention to engage in COVID-19 preventive behaviors were serially mediated through use of media channels for health communication and efficacious beliefs in both countries. The results imply that inequality in terms of both opportunities and experiences related to health communication can be a way in which we can address how socioeconomic inequalities have caused a decline in people’s willingness to engage in preventive behaviors in public health crises, as the SIM model posits [5, 23].

In particular, news media played a key mediating role in linking one’s socioeconomic positions and COVID-19 preventive behavioral intention. In the US and South Korea, news media use mediated the associations between the socioeconomic determinants of health, efficacy beliefs, and preventive behavioral intentions. One explanation for this finding is that news media might serve as gatekeepers to protect the population against the global infodemic which hampers efforts to contain the pandemic. News media can also contribute directly to citizens’ intention to engage in self-protection and enhance safety by debunking fake news and by providing trustworthy information and data regarding the virus [46]. Moreover, they can improve the efficiency of the governments’ pandemic response efforts [47]. However, unfortunately, the more socioeconomically disadvantaged a population was, the more difficult it was to receive the benefits of news media gatekeeping, which resulted in a lower willingness to comply with the suggested preventive behaviors during the COVID-19 pandemic.

Interestingly, use of instant messaging services for COVID-19 information played significantly different roles in shaping preventive behavioral intentions between the US and South Korea. Instant messenger usage was positively associated with self-efficacy in the US, but it surprisingly showed negative associations with response efficacy and preventive intention in South Korea. These contrasting findings imply that there may be cultural differences in use of instant messaging services between the US and South Korea, particularly with regard to COVID-19. According to cultural tightness-looseness theory, when it comes to controversial, sensitive social issues, people in countries with a tighter culture prefer to share their opinions regarding controversial issues only within their closed networks and with like-minded people [35]. In this regard, instant messaging services could be a channel that transmits misinformation and conspiracy theories about COVID-19 in South Korea [48–50]. As a result, frequent use of instant messaging services for COVID-19 information might lead to more frequent exposure to misinformation, which could, in turn, have a negative impact on efficacious beliefs and intentions regarding COVID-19 preventive measures in South Korea.

The use of instant messaging services and social media for health communication enhanced self-efficacy in the US but not in South Korea. The results for the US sample can be explained by the substitution argument [27, 28]. During the COVID-19 pandemic, Americans had limited interpersonal connections. According to a survey by the Pew Research Center [51], about one-in-five respondents in the US already worked from home all or most of the time before COVID-19, but this proportion increased to 71% in December 2020, during the pandemic outbreak, thereby reducing opportunities for interpersonal communication. Thus, Americans might use instant messaging services and social media to interact with others, which helped them build and improve self-efficacy with regard to COVID-19 preventive behaviors as substitutes for interpersonal interaction. However, in South Korea, where some level of social connection was maintained even during the pandemic due to the social, cultural, and environmental situations, such as the country’s high population density, lower popularity of working from home, and the use of public transportation.

It is noteworthy that both education and income were not directly associated with the intention to engage in COVID-19 preventive measures in either the US or South Korea. According to cumulative disadvantage theory, the effects of disadvantages in educational attainment and income accumulate over the life course, resulting in high levels of health inequality [52]. As socioeconomic status differences in health outcomes are not immediate but cumulative [53], predicting a significant relationship between socioeconomic status and preventive behavioral intention in the early stage of the COVID-19 pandemic may be unreasonable. Additionally, other potential confounding factors, along with education and income, could be significant predictors of preventive behavioral intention. We found that age, gender, and risk perception were directly related to the likelihood of engaging in preventive actions.

This research has significant theoretical and practical implications. Specifically, it contributes to theory elaboration by expanding the media channels for health communication from news media to online interactive media in the SIM framework. It also applies the SIM framework to countries with different culture based on the cultural tightness-looseness theory which is relevant to COVID-19 [34]. Practically, understanding what specific media result in socioeconomic inequalities with regard to preventive measures can aid in the prevention and control of global communicable diseases. In addition, the results of this study lend empirical support to the extant literature on health disparities and communication inequalities in public health crises. Our results indicate that health communication inequalities can mediate the effects of socioeconomic determinants of health on health outcomes during pandemics. Therefore, health policymakers and health communication researchers should develop educational interventions that enable especially socioeconomically disadvantaged groups to gain access to, understand, and apply health information from news media in times of public health crises. Our findings also reflect cross-cultural differences, suggesting that governments should consider adopting culture-driven policies and strategies for greater effectiveness. Given that health communication via instant messaging services had opposite associations with preventive behaviors during the COVID-19 pandemic in the US and South Korea, we suggest that the US government should actively utilize instant messaging services as an important communication channel during public health crises, while the South Korea government need to be cautious about potential unintended outcomes of instant messenger use for COVID-19 information (e.g., spread of misinformation) which might prevent citizens from engaging in preventive practices. In addition, household income played a more fundamental role than education in affecting media use for obtaining health information during the COVID-19 pandemic in South Korea. Thus, the South Korea government should also investigate and eliminate critical barriers that might hinder media use among economically vulnerable populations.

The present study has some limitations that need to be addressed. First, the cross-sectional nature of the data means it is limited in terms of confirming any causal claims in the proposed model. Second, we were only able to reach individuals who were enrolled as members of the online panels of research firms in each country. This purposive sampling method resulted in an oversampling of white Americans and Koreans with higher education, which, in turn, constrained the generalizability of the results. Third, the Cronbach’s alpha coefficient for news media use was low in the South Korea sample, which could be attributed to some of the items that may not adequately represent the dimension of news media use. Amid the COVID-19 pandemic, South Koreans exhibited distinct news consumption patterns for offline news media (e.g., television, radio, and newspapers) and online news media (e.g., internet news sites and news portals) [54]. As news media outlets serve different purposes and cater to diverse needs during a crisis [55], it is crucial to develop a valid and reliable questionnaire to measure the unique features of different types of news media. Fourth, our analysis using Harman’s single-factor test did not detect any common method variance (CMV), which is a severe methodological issue in survey research. However, Harman’s single-factor test is a diagnostic rather than a remedial approach to mitigate the impact of CMV on data quality [56]. Therefore, readers should interpret the results with caution. Finally, other potential predictors of preventive behavioral intention were excluded in this analysis, including occupation, neighborhood, social networks, and interpersonal communication, which may have led to biased estimates of associations. Future studies should attempt to examine the expanded framework of the SIM with additional constructs.

## Conclusions

To conclude, the present research is among the first efforts to investigate the mediating roles of health communication in the relationship between socioeconomic characteristics and preventive behavioral intention in the context of an infectious disease outbreak. The occurrence of indirect effects of household income and education on COVID-19 preventive actions through news media and instant messaging services as mediators can enrich future scholarship in the field of health disparities and health communication inequalities. Our findings highlight the need for up-to-date public health policies targeting socioeconomically vulnerable groups by facilitating the effective use of media for health information so as to prompt protective behaviors during pandemics and ultimately reduce health disparities.

## Data Availability

The datasets used and analyzed in the current study are available from the corresponding author on reasonable request.

## Abbreviations

COVID-19: Coronavirus disease 2019
SIM: Structural influence model
MLR: Maximum likelihood estimator with robust standard errors
RMSEA: Root mean square error of approximation
CFI: Comparative fit index
TLI: Tucker-Lewis index
SRMR: Standardized root mean square residual
